# Deep learning-based automated tongue analysis system for assisted Chinese medicine diagnosis

**DOI:** 10.3389/fphys.2025.1559389

**Published:** 2025-04-28

**Authors:** Tingnan Chen, Yutong Chen, Zili Zhou, Ying Zhu, Ling He, Jing Zhang

**Affiliations:** ^1^ College of Biomedical Engineering, Sichuan University, Chengdu, China; ^2^ Sichuan Second Hospital of TCM, Chengdu, China

**Keywords:** tongue images, image segmentation, image classification, tongue diagnosis, neural networks, color correction

## Abstract

This study proposes an automated tongue analysis system that combines deep learning with traditional Chinese medicine to enhance the accuracy and objectivity of tongue diagnosis. The system includes a hardware device to provide a stable acquisition environment, an improved semi-supervised learning segmentation algorithm based on U2net, a high-performance colour correction module for standardising the segmented images, and a tongue image analysis algorithm that fuses different features according to the characteristics of each feature of the TCM tongue image. Experimental results demonstrate the system’s performance and robustness in feature extraction and classification. The proposed methods ensure consistency and reliability in tongue analysis, addressing key challenges in traditional practices and providing a foundation for future correlation studies with endoscopic findings.

## 1 Introduction

The diagnosis of the tongue in traditional Chinese medicine is a method used to assess the health of internal organs by observing characteristics such as shape, color, texture, and coating of the tongue. However, traditional tongue diagnosis relies heavily on the experience of doctors, making it susceptible to subjective biases. With advancements in artificial intelligence and medical imaging technologies, intelligent tongue diagnosis systems based on image processing have emerged, providing an objective and standardized approach to tongue analysis. Nevertheless, due to variations in the photographic environment and lighting conditions, the tongue images of the same patient can differ significantly. Therefore, ensuring standardization in image acquisition and achieving precise image processing are key research challenges in the field of tongue diagnosis.

This paper proposes a deep learning-based tongue analysis system that uses image processing techniques to achieve automated segmentation and analysis of tongue images, ensuring stability and consistency in results. Specifically, we propose a semi-supervised learning-based tongue segmentation algorithm built on the U2Net model, incorporating several innovative modules for precise feature extraction and evaluation. The key innovations in this paper are as follows: (1) Development of a stable hardware and software environment for tongue image acquisition, ensuring standardized and rational image assessment. (2) An improved semi-supervised method based on the U2Net model, which effectively captures the scale characteristics of tongue segmentation and enhances model performance and generalization by utilizing a large number of unlabeled images. (3) A precise color correction method for the tongue image acquisition device, facilitating more standardized and accurate classification in subsequent analysis. (4) A comprehensive tongue diagnosis framework based on the image characteristics of different tongue types, achieving excellent performance even in challenging classification scenarios.

## 2 Related work

Tongue diagnosis has a long history in traditional Chinese medicine. With advancements in technology, digital image processing methods for tongue diagnosis have gradually developed. In recent years, many researchers have attempted to apply tongue image segmentation, color correction, and feature extraction in automated tongue diagnosis systems.

Existing tongue segmentation methods primarily include traditional image processing techniques and deep learning models. Early studies utilized edge detection and color thresholding methods for tongue segmentation; however, these approaches performed poorly in complex backgrounds. With the development of deep learning, convolutional neural networks (CNNs) have become the main tools for tongue segmentation. Kang et al. (2024) cascaded YOLOv5 with the LA-Unet network to refine the segmentation of tongue regions, optimizing segmentation for mobile tongue images. Zhang et al. (2022b) performed structural optimization on the DeeplabV3+ network, leveraging prior knowledge of tongue images to enhance edge regions, achieving precise results. Gao et al. (2021) proposed a level set model with symmetry and edge constraints, combining geometric features of the tongue for segmentation, capable of handling tongue images in most conditions. However, these segmentation methods only generalized their training to different imaging devices, making it challenging to achieve precise classification and recognition of tongue images, while not fully utilizing unlabeled tongue images. In other areas of biomedicine, Zhong et al. (2022) unsupervised approach to deconvolution in genomic subclones. Zhang et al. (2022a) proposing a lesion-aware dynamic network (LDNet) for polyp segmentation, which is a conventional U-shaped encoder-decoder structure combined with a dynamic kernel generation and update scheme. Gao et al. (2022) proposed a novel weak semi-supervised framework called SOUSA (Segmentation Using Only Sparse Annotations), which aims to learn from a small number of sparsely annotated datasets and a large amount of unlabeled data. Zhao et al. (2022) propose a cross-level contrast learning scheme to enhance the representation of local features in semi-supervised medical image segmentation. Inspired by these previous studies and given the scarcity of specialized tongue images, this paper proposes an improved U2Net tongue segmentation model combined with semi-supervised learning, enabling high-precision segmentation of tongue images captured by professional equipment, with strong interference resistance and robustness against abnormal tongue conditions. This approach effectively addresses the fragmentation of existing tongue analysis methods, enhancing their practical application value.

Furthermore, in terms of color correction for tongue images, existing research typically employs color mapping methods to address color discrepancies caused by different shooting devices and lighting conditions. Sun et al. Xin et al. (2021) proposed a gray world-based rapid color correction method for tongue images, assessing the degree of distortion after image compression, followed by color correction based on this degree, improving the effectiveness of color correction. Yan et al. (2021) introduced a TCCGAN network to correct tongue image colors, initially employing a differentiable weighted histogram network for color feature extraction, utilizing a new upsampling module called mixed feature attention upsampling to assist in image generation, while constructing a stacked network to generate tongue images from coarse to fine. However, enhancing the generalization ability of color correction methods to adapt to complex clinical environments remains a challenge. This paper proposes a space-distance-weighted Lasso regression algorithm, optimized for the regression environment of each color, effectively addressing issues such as image distortion and color overfitting after correction, laying a solid foundation for subsequent analysis of tongue and coating colors.

In tongue classification tasks, researchers have traditionally relied on texture feature extraction and machine learning classifiers for tongue evaluation. Recently, with the widespread application of deep learning models, significant advancements have been made in neural network-based tongue feature extraction and classification models. Chen et al. (2023) employed the K-means algorithm for coating separation and utilized RGB components of images to assess tongue color, achieving a balance between simplicity and accuracy. Yiqin (2022) used a Gaussian mixture model to separate tongue coating from the tongue body, developing a model for tongue image restoration, and ultimately achieved good results in classifying tongue textures based on ResNet101. Zhang et al. (2023) implemented a dual-threshold segmentation method based on HSI color space to automatically extract tongue bodies from original tongue images, categorizing tongues into those with and without coating, and further classifying coating thickness based on area. In the biomedical field, there are also researchers who focus on image detail features using target detection methods, Zhao et al. Prisilla et al. (2023) identified which YOLO models (YOLOv5, YOLOv6, and YOLOv7) performed well in detecting LDH in different regions of the lumbar disc. However, the complex boundaries and diverse texture information in tongue images continue to pose challenges for models. Thus, designing models that address the complex features of tongue coatings has become a current research focus. This paper presents a tongue feature judgment module based on different tongue feature groupings and precise coating separation, employing a precisely annotated coating separation dataset and a high-performance GSCNN model capable of handling complex boundaries, while utilizing LBP images and wavelet fusion features to significantly improve accuracy in difficult tongue classification.

Additionally, some researchers have combined other medical features with machine learning to implement end-to-end tongue applications. Yuan et al. (2023) integrated tongue coating microbiomes to establish an AI deep learning model, evaluating the value of tongue images and microbiomes in gastric cancer diagnosis. In other areas of biomedicine, Syed (2023) develop an automatic deep learning-based brain atrophy diagnosis model to detect, segment, classify, and predict the survival rate. Alabi et al. (2022) discussed deep learning technical knowledge and algorithms for OSCC and the application of deep learning techniques to cancer detection, image classification, segmentation and synthesis, and treatment planning. Zhou et al. (2022) summarized the workflows of deep learning methods in medical images and the current applications of deep learning-based AI for diagnosis and prognosis prediction in bone tumors. Having received help in working with the above systems, this paper proposes a comprehensive tongue diagnosis system that integrates tongue image acquisition, segmentation, color correction, and judgment, allowing accurate and efficient capture of key features of patients’ tongue images.

## 3 Clinical samples

The clinical samples in this study were obtained from 2,738 patients at Sichuan Province Second Hospital of Traditional Chinese Medicine, Mianzhu City Traditional Chinese Medicine Hospital, Guanghan City Traditional Chinese Medicine Hospital, and Anyue County People’s Hospital. Tongue images of these patients were collected using a specially designed tongue imaging device, and they underwent gastrointestinal endoscopy. Ethics approval for this study was obtained from Medical Ethics Committee of Sichuan Second Hospital of Traditional Chinese Medicine [approval number: 202304(H)-003-01]. All patients signed informed consent forms, agreeing to the use of their clinical samples for this research, and provided personal information including name, age, ethnicity, occupation, medical history and use of medications.

The tongue image data for each patient, including tongue body area, tongue coating area, tongue color, tongue texture (including tooth-marked tongue and cracked tongue), tongue shape, coating color, and coating texture, were annotated by professional physicians from the aforementioned hospitals. Due to the complex boundaries of the tongue coating area, pixel-level annotation was challenging for regular annotation tools; therefore, high-standard pixel-level annotations were conducted using Photoshop.

The tongue color was categorized into five types: pale, light red, red, dark red, and others. The coating color was classified into white, yellow, and other types. Additionally, various tongue conditions were classified as follows: tooth-marked tongue (presence or absence), cracked tongue (presence or absence), thickness (thick or thin coating), spotted tongue (presence or absence), peeling coating (presence or absence), curdy or slimy coating (curdy coating, slimy coating, or normal coating), and moist-dry condition (slippery coating, moist coating, or dry coating).

## 4 Materials and methods

**FIGURE 1:**
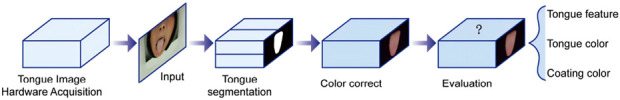
Flowchart of the intelligent tongue diagnosis system.

A complete tongue image segmentation and evaluation system was established. As shown in Figure 1, the system consists of four modules: tongue image acquisition hardware module, segmentation module, color correction module, and evaluation module.

### 4.1 Tongue image acquisition hardware module

**FIGURE 2:**
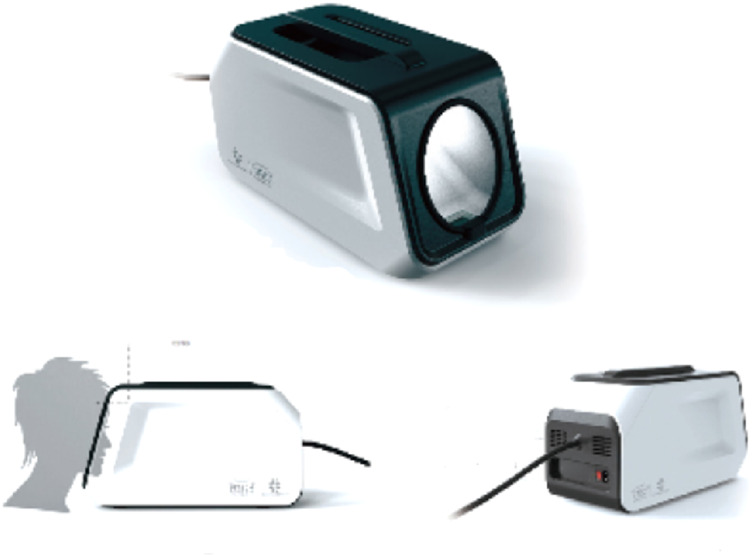
Appearance of the structure of the tongue filming equipment.

As shown in Figure 2,The acquisition box provides a constant light source to stabilise the tongue image acquisition environment, a customised industrial camera is used to provide high quality images, and the box is designed with a tilted angle to facilitate the presentation of the patient’s tongue. A mandibular rest is also used for the purpose of positioning the tongue.

### 4.2 Tongue image segmentation module

A semi-supervised learning algorithm based on the U2Net model is proposed to address the characteristics of tongue images. In the acquired tongue images, most of the pixels are occupied by the subject’s face and tongue, and the segmentation involves only the foreground and background, making it a salient object detection (SOD) task. Previous studies have used conventional segmentation networks like Mask R-CNN and DeepLabv3+ for tongue segmentation. Although these networks achieve excellent segmentation performance, their pre-trained backbone networks are typically trained on ImageNet, which limits their performance on specific tongue image segmentation tasks. To overcome these issues, we used U2Net as the main segmentation model structure to achieve stability in this SOD task for tongue segmentation. Since pixel-level labeling of tongue images requires substantial time and effort, we adapted it to a semi-supervised model to make full use of the data and achieve optimal training results.

U2Net (U-square-Net) Qin et al. (2020) is a deep learning-based segmentation model particularly suitable for salient object detection and segmentation tasks. Its name comes from its unique nested U-shaped structure. This model improves upon the traditional U-Net by incorporating several smaller U-Nets (called Residual U-blocks or RSU modules) to enhance feature extraction and multi-scale feature aggregation capabilities. The overall model consists of a U-shaped structure with 11 stages, each containing an RSU module, forming a six-level encoder and a five-level decoder, with skip connections between corresponding encoder and decoder layers to fuse multi-scale features.

To better suit the characteristics of tongue images, we adapted U2Net into a semi-supervised model called U2Net-MT. Mean Teacher Tarvainen and Valpola (2017) is a deep learning model for semi-supervised learning, in which a teacher network and a student network are used. The parameters of the teacher network serve as the targets for the student network, allowing the student to gradually learn more accurate parameters. This method effectively utilizes unlabeled data and enhances model generalization, especially in scenarios with limited labeled data.

**FIGURE 3:**
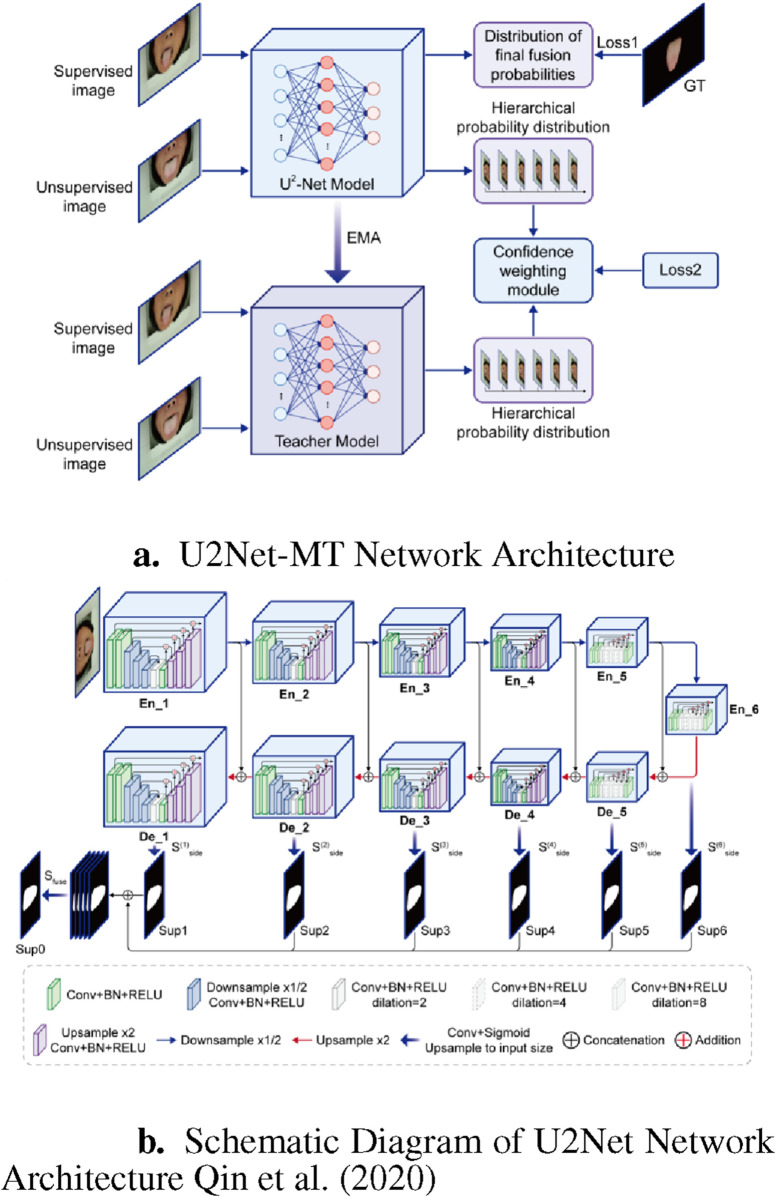
U2Net-MT Network. **(a)** U2Net-MT Network Architecture. **(b)** Schematic Diagram of U2Net Network Architecture Qin et al. (2020).

As shown in Figure 3, during training, both labeled tongue image A and unlabeled tongue image B are input into both the U2Net student model and the teacher model (which share the same structure). Random noise and image augmentations (including horizontal flipping and random cropping) are added to the different inputs in each model.

#### 4.2.1 Encoder and decoder

In the U2Net model, the input image I is processed by the encoder to generate a set of hierarchical features $M=\left\{m_{1},m_{2},\ldots ,m_{6}\right\}$ . For each feature $m_{i}$ , the decoder processes it hierarchically. In the decoder, each level’s input is obtained by concatenating (using the Concat operation) the output of the lower-level decoder and the corresponding encoder output, expressed as Equation 1:

$$d_{i}=\text{Concat}\left(u_{i+1},m_{i}\right)$$ (1)

where $u_{i+1}$ is the output of the lower-level decoder, and $m_{i}$ is the output of the corresponding encoder. Each scale’s output $O_{i}$ in the decoder undergoes a 3×3 convolution followed by a sigmoid activation to generate the saliency probability map $S_{i}$ , given by Equation 2:

$$O_{i}=\sigma \left(W_{i}*d_{i}+b_{i}\right)$$ (2)

where $W_{i}$ represents the convolution kernel, ∗ denotes the convolution operation, $b_{i}$ is the bias term, and σ is the sigmoid activation function defined as Equation 3:

$$\sigma \left(x\right)=\frac{1}{1+e^{-x}}$$ (3)

The output $O_{i}$ is upsampled to match the input image size, and then concatenated with the input image. After a 1×1 convolution and sigmoid activation, the final saliency probability map $S_{\text{fuse}}$ is obtained, given by Equations 4, 5:

$$O_{i}^{\text{up}}=\text{Upsample}\left(O_{i}\right)$$ (4)

$$S_{\text{fuse}}=\sigma \left(W_{\text{fuse}}*\text{Concat}\left(O_{i}^{\text{up}},I\right)+b_{\text{fuse}}\right)$$ (5)

#### 4.2.2 Loss function

For the labeled tongue image A , the loss $L_{1}$ is calculated for the fused probability map $S_{a}$ using the binary cross-entropy (BCE) loss (Equation 6):

$$L_{1}=-\frac{1}{N}\overset{N}{\underset{i=1}{\sum }}\left[y_{i}\log\left(S_{ai}\right)+\left(1-y_{i}\right)\log\left(1-S_{ai}\right)\right]$$ (6)

For all input images, saliency maps $S_{is}$ and $S_{it}$ are obtained for each level’s decoder from the student and teacher models, respectively. A loss function $L_{2i}$ is calculated at each level using the mean squared error (MSE) between $S_{is}$ and $S_{it}$ (Equation 7):

$$L_{2i}=\frac{1}{N}\overset{N}{\underset{j=1}{\sum }}{\left(S_{\mathrm{isj}}-S_{\mathrm{itj}}\right)}^{2}$$ (7)

A confidence weighting module is introduced to strengthen the loss function. The total consistency loss $L_{2}$ is computed by weighting each $L_{2i}$ with the average pixel confidence $Conf_{i}$ of $S_{is}$ and $S_{it}$ (Equation 8):

$$L_{2}=\underset{i}{\sum }\left(\frac{\omega_{i}L_{2i}}{\sum_{i}\omega_{i}}\right)$$ (8)

where $\omega_{i}$ is calculated as the average confidence of $S_{is}$ and $S_{it}$ . The overall loss of the model is given by (Equation 9):

$$L=L_{1}+\lambda L_{2}$$ (9)

where λ is the weighting coefficient for the loss. During training, L is minimized to obtain the final model parameters.

The trained student model is used for inference to evaluate its performance.

### 4.3 Color correction module

Equations should be inserted in editable format from the equation editor. In this experiment, all tongue images were captured using a mature, fixed hardware system. Therefore, all images undergo a strict color correction process to ensure data accuracy and consistency. During the experiment, ColorChecker 24 color card images were taken using different hardware devices of the same model, and various regression methods were used to calibrate the RGB values of the images to match the true values. Below, the basic theory of color correction and the applied color correction module are described.

#### 4.3.1 Basic principles of color correction

In color correction, the aim is to find the mapping relationship between the RGB values of the image and the standard color values of the ColorChecker 24 color card. Instead of performing regression on each color channel (R, G, B) separately, all RGB values are treated as a whole for regression analysis, which can account for correlations among RGB values and achieve more accurate color correction results.

##### 4.3.1.1 Color correction process

A multiple regression model is built, including RGB values, to directly map the original RGB values to the corrected RGB values. The regression model can be represented as Equation 10:

$$\left[\begin{array}{c} R_{\text{corrected}}\\ G_{\text{corrected}}\\ B_{\text{corrected}} \end{array}\right]=X\left[\begin{array}{c} \beta_{R}\\ \beta_{G}\\ \beta_{B} \end{array}\right]$$ (10)

where X is the design matrix containing the original RGB values, represented as $\left[\begin{array}{cccc} R & G & B & 1 \end{array}\right]$ . $\beta_{R},\beta_{G},\beta_{B}$ are the regression coefficients to be solved, which map the original RGB values to corrected RGB values. $R_{\text{corrected}},G_{\text{corrected}},B_{\text{corrected}}$ represent the corrected red, green, and blue channel values.

In matrix form, this can be expressed as Equation 11:

Y=Xβ (11)

where Y contains the corrected RGB values, represented as $\left[\begin{array}{ccc} R_{\text{corrected}} & G_{\text{corrected}} & B_{\text{corrected}} \end{array}\right]$ , and β is the regression coefficient matrix (Equation 12):

$$\left[\begin{array}{cccc} \beta_{R1} & \beta_{R2} & \beta_{R3} & \beta_{R0}\\ \beta_{G1} & \beta_{G2} & \beta_{G3} & \beta_{G0}\\ \beta_{B1} & \beta_{B2} & \beta_{B3} & \beta_{B0} \end{array}\right]$$ (12)

The regression coefficients are solved using the least squares method. Given n ColorChecker 24 samples and their standard values, the optimization problem can be expressed as Equation 13:

$$\min_{\beta }\overset{n}{\underset{i=1}{\sum }}{\left\|Y_{\text{true},i}-X_{i}\beta \right\|}^{2}$$ (13)

where $Y_{\text{true},i}$ is the true RGB value of the i -th sample, and $X_{i}$ is the original RGB value matrix of the i -th sample.

The best regression coefficients β can be solved using the matrix Equation 14:

$$\beta ={\left(X^{T}X\right)}^{-1}X^{T}Y_{\text{true}}$$ (14)

Using the obtained regression coefficients β , each pixel of a new image can be corrected. The steps are as follows:

For each pixel (R,G,B) , form an input vector (Equation 15):

$$X=\left[\begin{array}{cccc} R & G & B & 1 \end{array}\right]$$ (15)

Use the regression model to calculate the corrected RGB values (Equation 16):

$$Y_{\text{corrected}}=X\beta$$ (16)

Obtain the corrected RGB values $\left(R_{\text{corrected}},G_{\text{corrected}},B_{\text{corrected}}\right)$ .

#### 4.3.2 Lasso regression algorithm based on spatial distance weighting

ColorChecker 24 includes 24 color patches, covering a limited range of the color spectrum. This limitation may lead to overfitting issues, and the mapping derived from fitting these 24 colors often lacks generalization, making it less effective for correcting colors outside the range of the color patches.

To address this issue, this work employs a lasso regression algorithm based on spatial distance weighting.

##### 4.3.2.1 Calculating the distance and weight from pixels to ColorChecker 24

Suppose the RGB values of ColorChecker 24 in the XYZ Cartesian coordinate system are labeled as $CC_{n}\left(R_{n},G_{n},B_{n}\right)$ , where n=1,2,…,24 .

**FIGURE 4:**
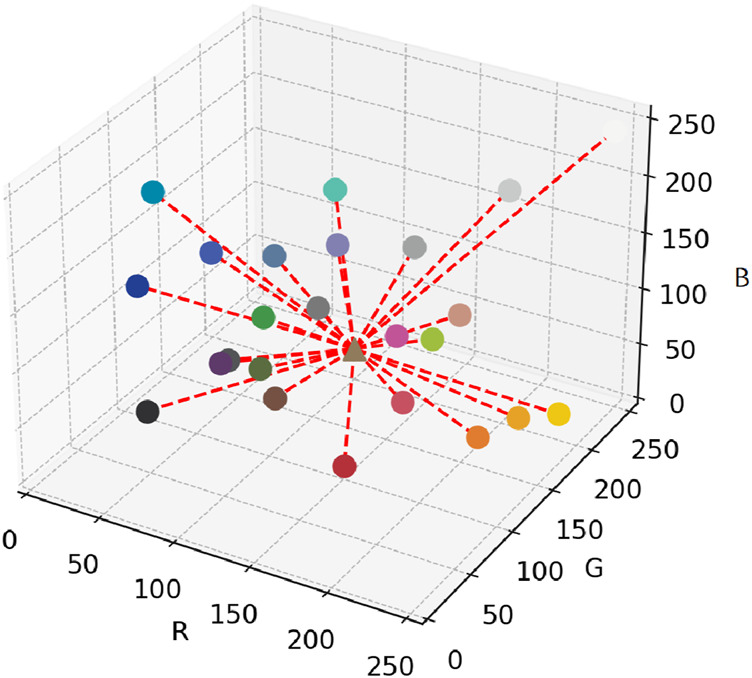
Spatial distribution of ColorChecker 24 patches and Euclidean distances to the target pixel.

As shown in Figure 4 for each pixel P(R,G,B) in the image, calculate its Euclidean distance to each $CC_{n}$ (Equation 17):

$$L_{n}=\sqrt{{\left(R-R_{n}\right)}^{2}+{\left(G-G_{n}\right)}^{2}+{\left(B-B_{n}\right)}^{2}}$$ (17)

Based on the calculated distance $L_{n}$ , determine the regression weight of each ColorChecker point $CC_{n}$ for point P (Equation 18):

$$w_{n}=\frac{1}{L_{n}}+\omega$$ (18)

where ω is a dilution term to prevent excessively large weights when $L_{n}$ is small.

##### 4.3.2.2 Polynomial feature transformation and lasso regression

Assume the degree of polynomial transformation is D . For each $CC_{n}$ and P , the transformed feature vector is Equation 19:

$$\begin{array}{cc} \text{Poly}\left(CC_{n}\right) & =\left[1,R_{n},G_{n},B_{n},R_{n}^{2},R_{n}G_{n},R_{n}B_{n},\right.\\ & \left.\;G_{n}^{2},G_{n}B_{n},B_{n}^{2},\ldots ,R_{n}^{D},G_{n}^{D},B_{n}^{D}\right]\\ \text{Poly}\left(P\right) & =\left[1,R,G,B,R^{2},RG,RB,\right.\\ & \left.\;G^{2},GB,B^{2},\ldots ,R^{D},G^{D},B^{D}\right] \end{array}$$ (19)

The weighted lasso regression model is then used to fit the data (Equation 20):

$$F\left(P\right)=\text{Lasso}\left(\alpha =0.01\right)\left(P\right)$$ (20)

The loss function of the model is (Equation 21):

$$\frac{1}{2m}\overset{m}{\underset{i=1}{\sum }}w_{i}{\left(y_{i}-\hat{y_{i}}\right)}^{2}+\alpha \overset{n}{\underset{j=1}{\sum }}|\theta_{j}|$$ (21)

where $w_{i}$ is the sample weight, $y_{i}$ is the actual value, $\hat{y_{i}}$ is the predicted value, α is the regularization parameter, and $\theta_{j}$ are the regression coefficients.

Use the trained lasso model to predict the corrected value for each pixel, and combine the corrected pixel values into the corrected image.

### 4.4 Tongue image analysis module

**FIGURE 5:**
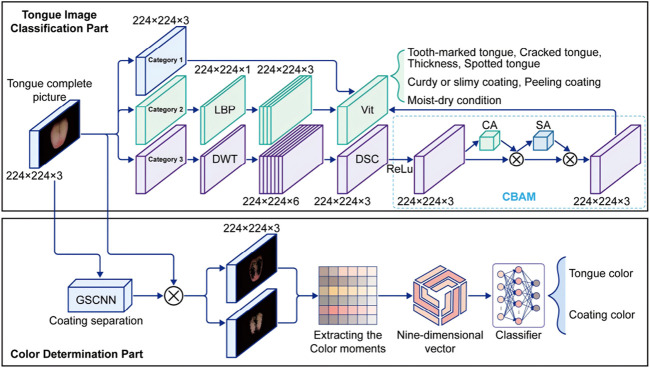
The network structure of the tongue image analysis module.

The tongue image analysis module consists of the tongue coating color determination module and the coating texture determination module. As shown in Figure 5, the color-corrected segmented tongue image is first input into the tongue coating color determination module. Here, the coating texture is separated into tongue coating and tongue body images, and the respective tongue and coating colors are determined. Finally, the color-corrected segmented tongue image is input into the coating texture determination module to obtain the texture analysis results.

#### 4.4.1 Color determination module

A deep learning model is used to separate the segmented tongue image into the coating and body images. Due to the complex boundaries and pixel-level distribution of tongue coatings, Gated-SCNN (Gated Shape Convolutional Neural Network) is introduced for coating separation.

Gated-SCNN (Gated Shape CNN) Takikawa et al. (2019) is an improved convolutional neural network architecture specifically designed for image segmentation tasks. Its main innovation is the introduction of a shape stream and gating mechanism. Structurally, Gated-SCNN consists of two parallel branches: the backbone network, responsible for extracting semantic features from the image, typically using ResNet or VGG; and the shape stream, which focuses on capturing edges and contours through a shape convolution module to extract multi-scale shape features. The shape information is fused with the semantic features using the gating mechanism, which dynamically adjusts the weight of the shape information at different locations. This combination enhances the ability of the model to handle complex boundaries and details, significantly improving segmentation accuracy.

After applying GSCNN to the input image, the result is inverted with the non-black regions of the original image to obtain the tongue body image.

For color determination of the tongue body and coating, color moments are used as feature extraction methods. Color moments Stricker and Orengo (1995) include the mean, variance, and skewness of color components. For each color component (e.g., red, green, and blue), the following three statistics are extracted:

1. Mean 2. Variance 3. Skewness.

These features are arranged in a specific order to form a nine-dimensional vector representing the color characteristics. Specifically, given a color component C , its mean, variance, and skewness are calculated as follows (Equations 22–24):

$$\mu_{C}=\frac{1}{N}\overset{N}{\underset{i=1}{\sum }}C_{i}$$ (22)

$$\sigma_{C}^{2}=\frac{1}{N}\overset{N}{\underset{i=1}{\sum }}{\left(C_{i}-\mu_{C}\right)}^{2}$$ (23)

$$\gamma_{C}=\frac{1}{N}\overset{N}{\underset{i=1}{\sum }}{\left(\frac{C_{i}-\mu_{C}}{\sigma_{C}}\right)}^{3}$$ (24)

Where $C_{i}$ represents the color value of the i -th pixel, N is the total number of pixels, $\mu_{C}$ is the mean, $\sigma_{C}^{2}$ is the variance, and $\gamma_{C}$ is the skewness of the color component.

The calculated mean, variance, and skewness are arranged in the order of red, green, and blue components to form a nine-dimensional feature vector (Equation 25):

$$F=\left[\mu_{R},\sigma_{R}^{2},\gamma_{R},\mu_{G},\sigma_{G}^{2},\gamma_{G},\mu_{B},\sigma_{B}^{2},\gamma_{B}\right]$$ (25)

This nine-dimensional vector F is then fed into a trained classifier to determine the tongue and coating color.

#### 4.4.2 Tongue image classification module

In the tongue image classification module, the features are divided into three categories:

Category 1: Teeth marks, cracks, thickness, and spots.

Category 2: Peeling and curdy or slimy.

Category 3: Moistness and dryness.

For Category 1, the features are clear and the deep learning network can easily extract them for direct classification. For Category 2, where peeling and curdy or slimy coatings are to be differentiated from non-peeling and normal coatings, the grayscale images are analyzed using Local Binary Pattern (LBP) operator for feature extraction.

The Local Binary Pattern (LBP) operator Ojala et al. (1994) is used for texture feature extraction. Given an input image I with a pixel at (x,y) :

The LBP value at each pixel is calculated as follows (Equation 26):

$$\text{LBP}\left(x,y\right)=\overset{P-1}{\underset{p=0}{\sum }}s\left(I\left(x_{p},y_{p}\right)-I\left(x,y\right)\right)\cdot 2^{p}$$ (26)

Where $\left(x_{p},y_{p}\right)$ are the neighboring pixels of (x,y) , P is the number of neighbors, and s(x) is a sign function defined as Equation 27:

$$s\left(x\right)=\left\{\begin{array}{cc} 1\; & \text{if }x\geq 0\\ 0\; & \text{if }x<0 \end{array}\right.$$ (27)

The LBP values for each pixel are stored at the corresponding location in the output image $I_{\text{LBP}}$ (Equation 28):

$$I_{\text{LBP}}\left(x,y\right)=\text{LBP}\left(x,y\right)$$ (28)

Where $I_{\text{LBP}}$ is of the same size as the original image I .

For Category 3, which deals with subtle texture changes, neural networks alone are insufficient. It was found experimentally that a combination of wavelet transformation and deep learning improved performance for distinguishing between moist and dry coatings.

In this experiment, the Daubechies wavelet (db3) was selected as the wavelet basis function. After the wavelet transformation, three high-frequency detail images are obtained: horizontal details (CH), vertical details (CV), and diagonal details (CD). To ensure the resolution matches that of the original image ( 224×224 pixels), interpolation was applied to these detail images.

The original tongue image $I\in R^{H\times W\times C}$ , where H and W represent the image height and width respectively, and C is the number of color channels. After wavelet transformation (Equation 29):

$$I_{CH},I_{CV},I_{CD}=\text{DWT}\left(I,\text{db}3\right)$$ (29)

Where DWT represents discrete wavelet transform and db3 is the wavelet basis.

Each detail image is resized back to 224×224 resolution (Equations 30–32):

$$I_{CH}=\text{Interp}\left(I_{CH},\left(224,224\right)\right)$$ (30)

$$I_{CV}=\text{Interp}\left(I_{CV},\left(224,224\right)\right)$$ (31)

$$I_{CD}=\text{Interp}\left(I_{CD},\left(224,224\right)\right)$$ (32)

Where Interp represents interpolation.

Next, the processed high-frequency detail images $I_{CH}$ , $I_{CV}$ , and $I_{CD}$ are concatenated with the original color channels $I_{R}$ , $I_{G}$ , and $I_{B}$ along the channel dimension, resulting in a concatenated image with six channels $I_{\mathrm{stacked}}$ (Equation 33):

$$I_{\mathrm{stacked}}=\left[I_{R},I_{G},I_{B},I_{CH},I_{CV},I_{CD}\right]$$ (33)

To achieve feature fusion, Depthwise Separable Convolution Howard et al. (2017) was used. Depthwise convolution applies a 3×3 convolution kernel to each input channel independently, as shown by Equation 34:

$$Y_{d}=I_{\text{stacked}}*K_{d}$$ (34)

where $Y_{d}$ represents the output after depthwise convolution, $K_{d}$ is the depthwise convolution kernel, and ∗ denotes the convolution operation.

Subsequently, pointwise convolution uses a 1×1 convolution kernel to combine information across all input channels (Equation 35):

$$Y_{p}=Y_{d}*K_{p}$$ (35)

where $Y_{p}$ is the output after pointwise convolution, and $K_{p}$ is the pointwise convolution kernel.

After the convolution operations, a ReLU activation function is applied (Equation 36):

$$Z=\text{ReLU}\left(Y_{p}\right)$$ (36)

To further enhance feature representation, the Convolutional Block Attention Module (CBAM) Woo et al. (2018) was integrated. CBAM first performs adaptive max pooling and adaptive average pooling on the input feature map to aggregate global information along the channel dimension, producing two descriptors (Equations 37, 38):

$$\text{MaxPool}\left(Z\right)=\text{Max}\left(Z,\text{dim}=H\times W\right)$$ (37)

$$\text{AvgPool}\left(Z\right)=\text{Avg}\left(Z,\text{dim}=H\times W\right)$$ (38)

These descriptors are then fed into shared convolutional layers consisting of two 1×1 convolution layers. The first convolutional layer reduces the number of channels to half of the original size and uses a ReLU activation function (Equation 39):

$$F_{1}=\text{ReLU}\left(W_{1}*\left[\text{MaxPool}\left(Z\right),\text{AvgPool}\left(Z\right)\right]\right)$$ (39)

The second convolutional layer restores the original channel size (Equation 40):

$$F_{2}=\sigma \left(W_{2}*F_{1}\right)$$ (40)

where σ represents the Sigmoid activation function. The output of the convolutional layers serves as channel attention weights, which are combined with the original feature map through element-wise multiplication to enhance important features.

The enhanced feature map is then processed by the spatial attention module. This module performs global max pooling and global average pooling along the spatial dimension to generate two single-channel feature maps (Equations 41, 42):

$${\text{MaxPool}}_{s}\left(Z\right)=\text{Max}\left(Z,\text{dim}=C\right)$$ (41)

$${\text{AvgPool}}_{s}\left(Z\right)=\text{Avg}\left(Z,\text{dim}=C\right)$$ (42)

These feature maps are concatenated along the channel dimension to form a two-channel feature map, which is then processed by a 7×7 convolution layer to generate the spatial attention map (Equation 43):

$$F_{s}=\sigma \left(W_{s}*\left[{\text{MaxPool}}_{s}\left(Z\right),{\text{AvgPool}}_{s}\left(Z\right)\right]\right)$$ (43)

The spatial attention map is combined with the channel-enhanced feature map through element-wise multiplication to further improve feature representation.

For the image classification task, Vision Transformer (ViT) Dosovitskiy et al. (2021) was used as the classification head. ViT divides the input image into fixed-size patches, flattens each patch, and embeds it into a high-dimensional vector space. These embedding vectors are then added to positional encodings to retain positional information and processed through multiple Transformer encoder layers.

The input image $Z\in R^{H\times W\times C}$ is divided into N patches of size P×P , where $N=\frac{HW}{P^{2}}$ . Each patch $z_{i}\in R^{P\times P\times C}$ is flattened and mapped to a high-dimensional vector through a linear transformation (Equation 44):

$$e_{i}=\text{Flatten}\left(z_{i}\right)W_{e}+b_{e}$$ (44)

where $W_{e}\in R^{\left(P^{2}C\right)\times D}$ is the embedding matrix, $b_{e}\in R^{D}$ is the bias vector, and D is the embedding dimension.

The positional encoding vector $E_{\text{pos}}\in R^{N\times D}$ is added to the embedding vectors to retain positional information (Equation 45):

$$E_{0}=\left[e_{1};e_{2};\ldots ;e_{N}\right]+E_{\text{pos}}$$ (45)

where $E_{0}\in R^{N\times D}$ is the initial embedding representation.

The initial embedding representation $E_{0}$ is processed through L Transformer encoder layers. Each encoder layer includes a Multi-Head Self-Attention (MHSA) mechanism and a Feed-Forward Neural Network (FFN):

Multi-Head Self-Attention (Equation 46):

$$\text{MHSA}\left(E\right)=\left[{\text{head}}_{1};{\text{head}}_{2};\ldots ;{\text{head}}_{h}\right]W_{O}$$ (46)

where each attention head is defined as (Equation 47):

$${\text{head}}_{i}=\text{Attention}\left(EW_{i}^{Q},EW_{i}^{K},EW_{i}^{V}\right)$$ (47)

The attention calculation is (Equation 48):

$$\text{Attention}\left(Q,K,V\right)=\text{softmax}\left(\frac{QK^{T}}{\sqrt{d_{k}}}\right)V$$ (48)

where Q,K,V are the query, key, and value matrices, respectively, and $d_{k}$ is the dimension of the keys.

Feed-Forward Neural Network (Equation 49):

$$\text{FFN}\left(E\right)=\text{ReLU}\left(EW_{1}+b_{1}\right)W_{2}+b_{2}$$ (49)

where $W_{1},W_{2}$ are weight matrices and $b_{1},b_{2}$ are bias vectors.

The output of each Transformer encoder layer is represented as (Equations 50, 51):

$$E_{l+1}=\text{LayerNorm}\left(E_{l}+\text{MHSA}\left(E_{l}\right)\right)$$ (50)

$$E_{l+2}=\text{LayerNorm}\left(E_{l+1}+\text{FFN}\left(E_{l+1}\right)\right)$$ (51)

where LayerNorm represents the layer normalization operation.

After L Transformer encoder layers, the final feature representation $E_{L}$ is fed into the classification head. A class token is introduced in the feature representation, which is processed by the Transformer encoder layers and used for the final classification task (Equation 52):

$$\text{class token}=E_{L}\left[0\right]$$ (52)

The classification is performed using a linear transformation followed by a softmax function (Equation 53):

$$y=\text{softmax}\left(W_{\text{cls}}\cdot \text{class token}\right)$$ (53)

where $W_{\text{cls}}$ is the weight matrix of the classification head.

Focal Loss was used as the loss function to handle the imbalance in tongue image features, calculated as (Equation 54):

$$\text{FL}\left(p_{t}\right)=-\alpha_{t}{\left(1-p_{t}\right)}^{\gamma }\log\left(p_{t}\right)$$ (54)

where $p_{t}$ represents the predicted probability, and $\alpha_{t}$ and γ are hyperparameters.

## 5 Experiments

### 5.1 Evaluation metrics

For the segmentation task, Mean Absolute Error (MAE) and the Dice coefficient were used as evaluation metrics. MAE provides an intuitive measure of the overall pixel-wise classification accuracy, while the Dice coefficient considers both precision and recall for all instances, focusing on the overlap with the target regions.

For the classification task, accuracy (acc) and macro-F1 score were used. This not only considers overall accuracy but also gives more weight to frequently occurring samples.

To evaluate the performance of color correction, $\Delta E_{ab}^{*}$ was used to quantify color differences. $\Delta E_{ab}^{*}$ is an index for quantifying and describing color differences, based on the CIELAB color space, and it compares the visual difference between two colors. A larger value of $\Delta E_{ab}^{*}$ indicates a more noticeable difference between the two colors. The calculation of $\Delta E_{ab}^{*}$ is as follows (Equation 55):

$$\Delta E_{ab}^{*}=\sqrt{{\left(\Delta L^{*}\right)}^{2}+{\left(\Delta a^{*}\right)}^{2}+{\left(\Delta b^{*}\right)}^{2}}$$ (55)

where:

• $\Delta L^{*}$ represents the difference in lightness $\left(L^{*}\right)$ between the two colors;
• $\Delta a^{*}$ represents the difference in the red-green axis $\left(a^{*}\right)$ between the two colors;
• $\Delta b^{*}$ represents the difference in the yellow-blue axis $\left(b^{*}\right)$ between the two colors.

### 5.2 Experimental results

#### 5.2.1 Performance analysis

To verify the performance of the proposed method, comparisons were made with existing methods used in tongue image segmentation and classification.

UNet Ronneberger et al. (2015) is a classic image segmentation network consisting of an encoder and a decoder, which effectively extracts and restores image details through its symmetric structure and skip connections. The encoder extracts features, the decoder restores resolution, and the skip connections transmit features between the encoder and decoder, preventing feature loss and improving segmentation accuracy. UNet has been widely used in medical image processing and biological image analysis. UNet++ Zhou et al. (2020) is an improved version of UNet, adding more dense skip connections and decoder submodules. It introduces additional convolution layers at each downsampling and upsampling stage to form dense connectivity paths, capturing multi-scale features and enhancing segmentation accuracy and robustness, which is suitable for segmenting complex image structures. FCN Long et al. (2014) (Fully Convolutional Network) removes the fully connected layers and only uses convolution and upsampling layers. It extracts features through a series of convolution layers and restores the original resolution through deconvolution. FCN’s design preserves spatial information and enables efficient pixel-level classification, suitable for end-to-end segmentation of images of any size. DeepLabv3+ Chen et al. (2018) combines the encoder-decoder structure and atrous convolution, using ResNet or Xception as the backbone. The encoder extracts multi-scale features, and the decoder enhances segmentation with atrous convolution to capture more contextual information. The multi-scale feature fusion strategy improves segmentation accuracy and detail retention, suitable for image segmentation in complex scenes. Mask R-CNN Massa and Girshick (2018) adds a branch for generating pixel-level segmentation masks on top of Faster R-CNN. The backbone extracts features, the region proposal network generates candidate regions, and each candidate is classified, regressed, and mask-generated. Mask R-CNN performs object detection and instance segmentation simultaneously, providing more precise segmentation results and being widely used in instance segmentation tasks. In this experiment, semi-supervised models used a 1:1 supervision rate, and GSCNN adopted VGG16 as the backbone, with all models loaded with pre-trained weights.

The Table 1 shows the comparison of U2net-MT and GSCNN with other segmentation networks on the dataset used in this study. Meanwhile Table 2 shows a more intuitive image of the segmentation results under different models. The results indicate that both networks achieved the best performance on two metrics, demonstrating that the proposed and applied methods effectively address the segmentation and coating separation problems of the tongue dataset. Although other models listed in the table have shown excellent performance in medical image processing and semantic segmentation, they lack focused analysis of features from specific depths during segmentation, and they struggle with ambiguous boundaries between the tongue, throat, and lips. For coating separation, traditional segmentation networks face challenges in extracting effective features from the highly detailed, dispersed, and weakly correlated coating on the tongue. U2net-MT combines U2net’s strengths in retaining full-resolution features and multi-scale feature fusion while optimizing significant target detection, with a semi-supervised approach to improve data utilization and generalization capability. By assigning more weight to high-confidence scales, the model enhances scale-specific attention during semi-supervised training, effectively improving the classification performance of fuzzy pixels around the tongue. GSCNN introduces the Canny operator in features, using a gating mechanism to ensure that only boundary-related information is processed in the shape stream, enabling it to effectively handle complex coating boundaries.

**TABLE 1:** Comparison of the effect of the model used in this experiment with other image segmentation model methods.

Model	Tongue segmentation		Coating separation	
	MAE	Dice	MAE	Dice
Unet	0.084	0.921	0.103	0.820
Unet++	0.045	0.946	0.069	0.854
DeeplabV3+	0.034	0.959	0.066	0.852
FCN	0.107	0.892	0.127	0.779
Mask-RCNN	0.061	0.938	0.076	0.846
U2net-MT/GSCNN	0.022	0.967	0.058	0.860

**TABLE 2:** Comparison of a complete tongue image and segmentation results in different models.

Original image	Unet	Unet++	Deeplab V3+	FCN	Mask-RCNN	U2net-MT
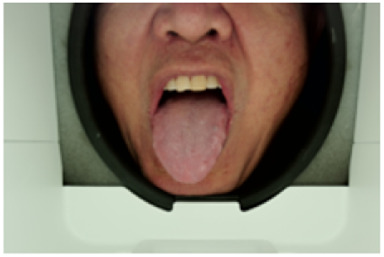	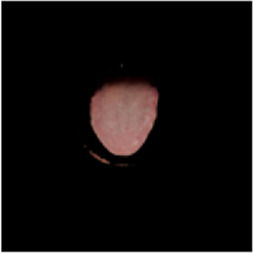	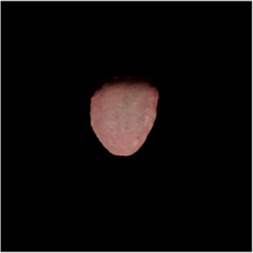	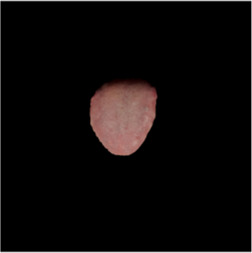	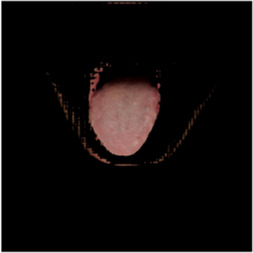	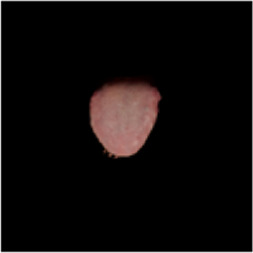	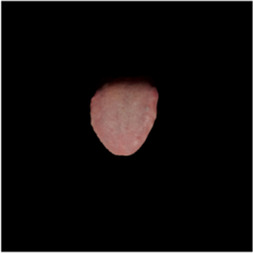

The Table 3 shows the results of the proposed spatial-distance-weighted Lasso regression algorithm and other common algorithms in color correction of the ColorChecker 24 color card, measured by $\Delta E_{ab}^{*}$ . The results show that the spatial-distance-weighted Lasso regression achieved the best results for both average and maximum/minimum $\Delta E_{ab}^{*}$ . This is because the spatial-distance-weighted Lasso regression focuses more on the regression relationships of similar colors instead of treating the 24 colors as a whole. Regularization via Lasso regression also effectively solves overfitting in small sample regressions. In contrast, traditional linear regression methods have poor accuracy in color correction, and higher-order regression, while adapting to the specific regression characteristics of each color, can have steep gradients at color boundaries, causing visual artifacts in the corrected images. These results indicate that spatial-distance-weighted Lasso regression effectively addresses subtle lighting variations caused by different environments, providing a basis for subsequent classification of tongue and coating colors.

**TABLE 3:** Comparison of Δ E*ab between the tongue colour correction method used in this paper and other commonly used colour correction methods.

Methods	Δ E*ab-ave	Δ E*ab-max
Origin image	10.60	22.55
LR (linear regression)	7.57	28.38
PR (Polynomial regression)	7.43	35.34
KNN	6.01	17.55
Adaboost	11.26	27.89
SVR	5.71	17.16
Ours-without Lasso (L1)	4.34	13.96
Ours-with Ridge (L2)	4.10	10.13
Ours	3.87	9.08

To ensure the classification results of tongue diagnosis are of the highest quality, different classifiers were applied to the Color and Tongue feature sections to observe the experimental outcomes. Due to space limitations in the table, only some of the more effective methods are selected for comparison. In the Color section, the classifiers primarily focus on the processed visual color features. As a result, simpler machine learning classification methods are more effective in the experiments. Among these methods, Random Forest (RF) demonstrates excellent anti-overfitting capabilities and can also filter the importance of features, thereby validating whether the extracted features contribute significantly to classification. Regarding K-Nearest Neighbors (KNN), the color-classification boundaries in these color matrix samples should be relatively clear, making KNN quite effective. Similarly, in the more complex color matrix vectors, Support Vector Machines (SVM) can maximize the margin between classes, and its advantage over KNN is that it can be trained in advance. The Softmax method, combined with a simple neural network structure, performs excellently in multi-class classification tasks and can also be optimized through backpropagation.

In the Tongue feature section, the classical ResNet structure is also used for comparison. The reason for choosing it is its ability to extract deep features from images while being highly versatile. EfficientNet enhances computational efficiency by optimizing the structure, offering good advantages in texture feature computation. MobileNet maintains high efficiency in lightweight design, and the reason for selecting it is to observe the performance of lightweight models in tongue feature extraction and computation. Vision Transformers (ViT), relying on the self-attention mechanism and Transformer architecture, are able to capture complex patterns and details in images, and they perform well on large-scale datasets. By combining the convolutional feature extraction capability of ResNet50 with the global self-attention mechanism of ViT, both local and global information can be utilized. If these features are useful, the classification performance will be enhanced. Among these methods, the focus of this paper is on those that demonstrate efficient and stable performance across various features.

The Table 4 presents the results of the proposed tongue diagnosis algorithm with different classifiers. The extracted tongue features allowed the classifiers to effectively classify based on tongue characteristics, indicating that tongue diagnosis, as an integrated module, could effectively extract overall tongue information of patients. Selecting the appropriate classifier for different information yielded better results. In experiments, the ViT model maintained stable and excellent performance in multiple classification tasks. This is because the relatively simple structure of the transformer and the efficient attention mechanism can focus on different texture classification features. Therefore, the ViT-b model was chosen for inference in the tongue diagnosis module. Additionally, the ViT with ResNet as the backbone did not perform as well in most classification tasks, indicating that deep feature extraction is limited for guiding classification. The performance of lightweight networks was also not remarkable, reaffirming the suitability of the transformer structure for texture features of the tongue.

**TABLE 4:** Performance of multiple models and classifiers in tongue image feature classification (%).

Part	Category	Methods	Acc	Macro-f1
Color	Tongue Color	RF	86.27	67.65
		KNN	91.36	79.21
		SVM	93.10	81.29
		Softmax	91.75	80.03
	Coating Color	RF	94.71	84.89
		KNN	97.30	86.97
		SVM	97.48	88.04
		Softmax	98.09	88.73
Tongue feature	Tooth-marked tongue	Resnet50	92.13	87.38
		EfficientNetV2	94.16	90.77
		MobileNetV2	85.26	84.39
		Resnet50+ViT	93.64	90.61
		ViT-b	96.77	93.20
	Cracked tongue	Resnet50	98.14	95.33
		EfficientNetV2	93.26	92.97
		MobileNetV2	87.13	85.01
		Resnet50+ViT	96.56	94.72
		ViT-b	98.65	95.19
	Thickness	Resnet50	85.18	82.53
		EfficientNetV2	85.57	83.09
		MobileNetV2	79.53	69.42
		Resnet50+ViT	86.45	83.22
		ViT-b	86.11	83.39
	Spotted tongue	Resnet50	98.07	97.15
		EfficientNetV2	98.76	98.04
		MobileNetV2	98.10	97.06
		Resnet50+ViT	98.23	97.21
		ViT-b	98.84	97.95
	Peeling coating	Resnet50	92.55	90.47
		EfficientNetV2	94.61	92.44
		MobileNetV2	90.73	82.39
		Resnet50+ViT	94.18	91.97
		ViT-b	94.32	92.58
	Curdy or slimy coating	Resnet50	81.20	74.53
		EfficientNetV2	87.66	80.30
		MobileNetV2	80.74	73.61
		Resnet50+ViT	92.95	83.88
		ViT-b	92.62	82.05
	Moist-dry condition	Resnet50	74.83	59.40
		EfficientNetV2	81.09	61.14
		MobileNetV2	69.95	39.62
		Resnet50+ViT	84.54	66.07
		ViT-b	86.61	71.20

#### 5.2.2 Ablation study

This section presents ablation experiments to validate the effectiveness of various components and key methods in each module of the tongue image system on the test set. “Without” indicates the absence of the specific key method from the module. The experiments are divided as follows: (1) Evaluation of the MT semi-supervised module and confidence-weighted module in U2net-MT on the noisy test set. (2) Evaluation of the effectiveness of the LBP features from the second group of tongue images, wavelet features from the third group, feature fusion, and the CBAM module in the tongue diagnosis module.

As shown in the Table 5, U2net-MT achieved optimal results for both metrics. Removing the MT semi-supervised method led to a significant decrease in the Dice coefficient, indicating that the MT semi-supervised method effectively enhances the model’s generalization capability, reduces the impact of noise, and improves accuracy by leveraging features from unlabeled data. Removing the confidence-weighted module resulted in a performance drop in both metrics, suggesting that the confidence-weighted module helps the model focus on feature information that is beneficial for tongue image segmentation during backpropagation. These findings demonstrate the effectiveness of the methods used in U2net-MT for training in tongue image segmentation.

**TABLE 5:** Experimental effects of ablation on each module of U2net-MT.

	MAE	Dice
U2net	0.030	0.956
U2net-MT without confidence weighting module	0.024	0.961
U2net-MT	0.022	0.967

The Table 6 shows the comparison results between the extracted features from the second and third classification groups and the original images used directly for tongue classification. For the peeling and greasy tongue coatings, which have significant texture differences in grayscale images, LBP feature extraction significantly improved classification performance by filtering out much irrelevant noise. In the third group, related to the moist-dry classification, the proportion of relevant features in the images was too low for the model to extract effective information for classification at various levels. The experiments demonstrated that wavelet transform, which provides time-frequency localization, could accurately capture subtle texture features in tongue images. The wavelet features effectively reflect the moist-dry correlation, solving the challenging moist-dry classification problem. The fusion of original image features added complementary detail features, further improving classification performance. Introducing CBAM channel and spatial attention mechanisms allowed the model to focus more on key features, enhancing classification accuracy.

**TABLE 6:** Results of ablation experiments on the strategy of using LBP and wavelet features in tongue image classification (%).

Using feature (ViT)	Acc	Macro-F1
Origin image of peeling/Curdy or slimy coating	80.40/85.50	59.71/70.27
LBP	94.32/92.62	92.58/82.05
Origin image of moist-dry condition	55.67	33.4
Origin image of moist-dry condition + Spectrogram	54.32	33.3
Wavelet Feature	84.40	65.88
Origin image of moist-dry condition + Wavelet Feature	86.61	71.20

### 5.3 Conclusion

In this study, a complete tongue image analysis system was successfully developed, combining modern deep learning techniques with traditional Chinese medicine tongue diagnosis to improve the accuracy of tongue segmentation and coating assessment. Specifically, the semi-supervised learning algorithm based on the U2Net model significantly improved the quality of image segmentation. In addition, the color correction module ensured the accuracy and consistency of image data, and wavelet features were integrated for tongue diagnosis analysis. Experimental results demonstrated the system’s outstanding performance in feature extraction and classification of tongue images. Furthermore, the color correction strategy effectively resolved color deviations caused by device differences and environmental variations, providing a more reliable foundation for tongue image analysis. The integration of wavelet features also effectively addressed the challenging problem of moist-dry classification. In future work, the relationship between patients’ tongue characteristics and endoscopic examination results will be analyzed to explore their correlation.

This work not only realizes automated tongue analysis, but also provides real-time feedback of the analysis results, reducing the time and effort required for manual diagnosis. This is important for improving diagnosis and treatment efficiency and reducing the workload of medical staff, especially in large-scale patient management and telemedicine.

## Data Availability

The original contributions presented in the study are included in the article/Supplementary Material, further inquiries can be directed to the corresponding author.
