# Supplementary material for: Deep learning-based automated tongue analysis system for assisted Chinese medicine diagnosis
Source: Front Physiol. 2025 Apr 28;16:1559389. doi: 10.3389/fphys.2025.1559389 (PMC12066954; doi:10.3389/fphys.2025.1559389)
Supplement: Supplementary file 1 [file DataSheet1.pdf]

frontiers

Supplementary Material

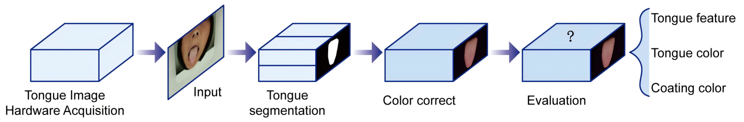

Figure S1. Flowchart of the intelligent tongue diagnosis system

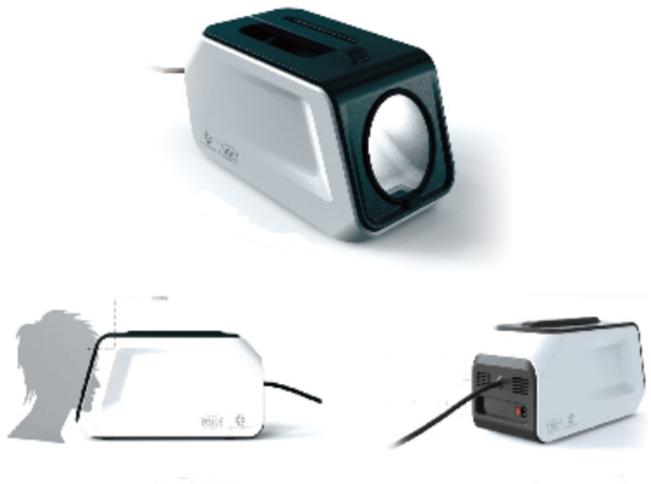

Figure S2. Appearance of the structure of the tongue filming equipment

Table S1. Comparison of the effect of the model used in this experiment with other image segmentation model methods

Model	Tongue segmentation		Coating separation	
	MAE	Dice	MAE	Dice
Unet	0.084	0.921	0.103	0.820
Unet++	0.045	0.946	0.069	0.854
DeeplabV3+	0.034	0.959	0.066	0.852
FCN	0.107	0.892	0.127	0.779
Mask-RCNN	0.061	0.938	0.076	0.846
**U2net-MT/GSCNN**	**0.022**	**0.967**	**0.058**	**0.860**

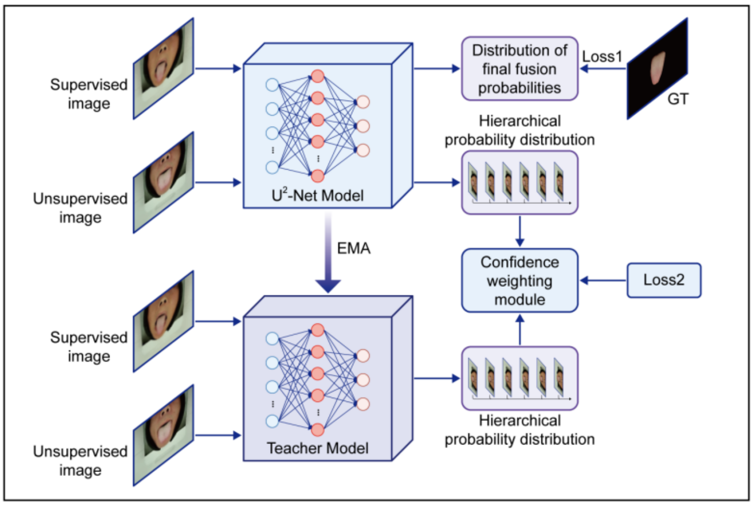

Figure S3. U2Net-MT Network Architecture

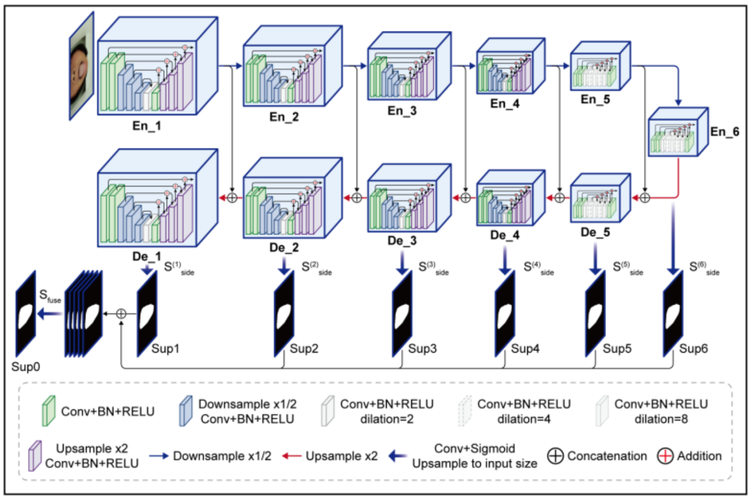

Figure S4. Schematic Diagram of U2Net Network Architecture

Table S2. Comparison of a complete tongue image and segmentation results in different models

Original image	Unet	Unet++	Deeplab V3+	FCN	Mask-RCNN	U2net-MT
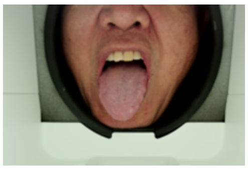	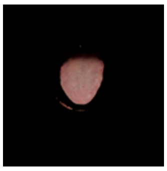	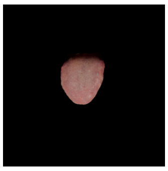	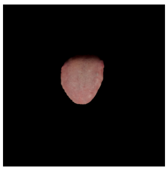	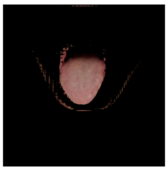	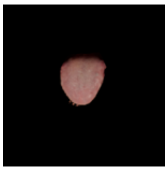	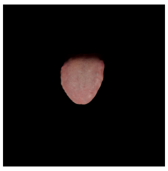

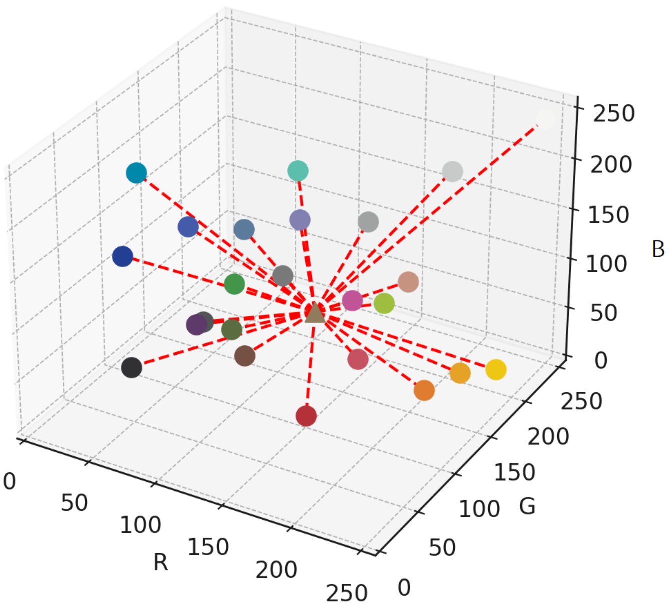

Figure S5. Spatial distribution of ColorChecker 24 patches and Euclidean distances to the target pixel

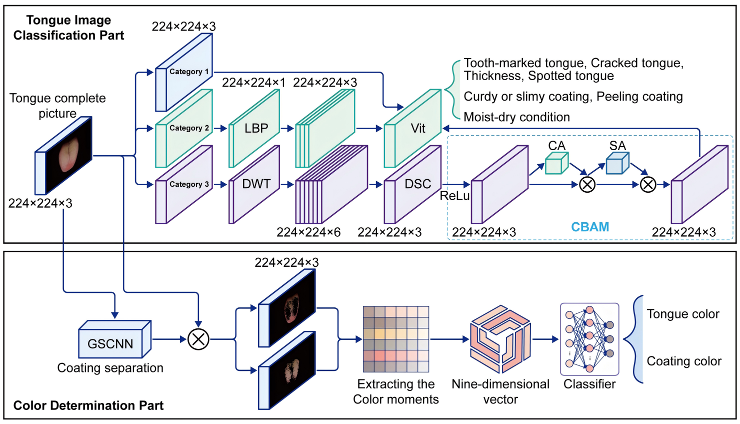

Figure S6. The network structure of the tongue image analysis module

Table S3. Comparison of ΔE*ab between the tongue colour correction method used in this paper and other commonly used colour correction methods

Methods	ΔE*ab-ave	ΔE*ab-max
Origin image	10.60	22.55
LR(linear regression)	7.57	28.38
PR(Polynomial regression)	7.43	35.34
KNN	6.01	17.55
Adaboost	11.26	27.89
SVR	5.71	17.16
Ours-without Lasso(L1)	4.34	13.96
Ours-with Ridge(L2)	4.10	10.13
**Ours**	**3.87**	**9.08**

Table S4. Performance of multiple models and classifiers in tongue image feature classification (%)

Part	Category	Methods	Acc	Macro-f1
Color	Tongue Color	RF	86.27	67.65
		KNN	91.36	79.21
		**SVM**	**93.10**	**81.29**
		Softmax	91.75	80.03
	Coating Color	RF	94.71	84.89
		KNN	97.30	86.97
		SVM	97.48	88.04
		**Softmax**	**98.09**	**88.73**
Tongue feature	tooth-marked tongue	Resnet50	92.13	87.38
		EfficientNetV2	94.16	90.77
		MobileNetV2	85.26	84.39
		Resnet50+ViT	93.64	90.61
		**ViT-b**	**96.77**	**93.20**
	cracked tongue	Resnet50	98.14	**95.33**
		EfficientNetV2	93.26	92.97
		MobileNetV2	87.13	85.01
		Resnet50+ViT	96.56	94.72
		ViT-b	**98.65**	95.19
	thickness	Resnet50	85.18	82.53
		EfficientNetV2	85.57	83.09
		MobileNetV2	79.53	69.42
		Resnet50+ViT	**86.45**	83.22
		ViT-b	86.11	**83.39**
	spotted tongue	Resnet50	98.07	97.15
		EfficientNetV2	98.76	**98.04**
		MobileNetV2	98.10	97.06
		Resnet50+ViT	98.23	97.21
		ViT-b	**98.84**	97.95
	peeling coating	Resnet50	92.55	90.47
		EfficientNetV2	**94.61**	92.44
		MobileNetV2	90.73	82.39
		Resnet50+ViT	94.18	91.97
		ViT-b	94.32	**92.58**
	Curdy or slimy coating	Resnet50	81.20	74.53
		EfficientNetV2	87.66	80.30
		MobileNetV2	80.74	73.61
		Resnet50+ViT	**92.95**	**83.88**
		ViT-b	92.62	82.05
	moist-dry condition	Resnet50	74.83	59.40
		EfficientNetV2	81.09	61.14
		MobileNetV2	69.95	39.62
		Resnet50+ViT	84.54	66.07
		**ViT-b**	**86.61**	**71.20**

Table S5. Experimental effects of ablation on each module of U2net-MT

	MAE	Dice
U2net	0.030	0.956
U2net-MT without confidence weighting module	0.024	0.961
**U2net-MT**	**0.022**	**0.967**

Table S6. Results of ablation experiments on the strategy of using LBP and wavelet features in tongue image classification (%)

Using Feature (ViT)	Acc	Macro-F1
Origin image of peeling/Curdy or slimy coating	80.40/85.50	59.71/70.27
LBP	**94.32/92.62**	**92.58/82.05**
Origin image of moist-dry condition	55.67	33.4
Origin image of moist-dry condition +Spectrogram	54.32	33.3
Wavelet Feature	84.40	65.88
Origin image of moist-dry condition + Wavelet Feature	**86.61**	**71.20**
